# Healthcare-associated infections and antimicrobial resistance in severe acquired brain injury: a retrospective multicenter study

**DOI:** 10.3389/fneur.2023.1219862

**Published:** 2023-08-16

**Authors:** Giovanna Barbara Castellani, Elisa Maietti, Gloria Leonardi, Erik Bertoletti, Filippo Trapani, Alberto Battistini, Sara Tedeschi, Roberto Piperno, Pierluigi Viale

**Affiliations:** ^1^Montecatone Rehabilitation Institute, Imola, Italy; ^2^Department of Biomedical and Neuromotor Sciences, University of Bologna, Bologna, Emilia-Romagna, Italy; ^3^Department of Long-Term Care Rehabilitation, Santa Viola Hospital Colibrì Consortium, Bologna, Italy; ^4^Infectious Disease Unit, Department of Integrated Management of Infectious Risk, IRCCS Azienda Ospedaliero-Universitaria di Bologna, Bologna, Italy; ^5^Rehabilitation Medicine and Neurorehabilitation Unit, IRCCS Istituto delle Scienze Neurologiche di Bologna, Bologna, Italy; ^6^Department of Medical and Surgical Sciences, University of Bologna, Bologna, Italy

**Keywords:** rehabilitation outcome, brain injuries, infections, multidrug resistance, carbapenemase-producing Enterobacteriaceae

## Abstract

**Background:**

Recent studies underscore that healthcare-associated infections (HAIs) and multidrug-resistant (MDR) HAIs affect rehabilitation outcomes and hospital length of stay (LOS) for severe acquired brain injury (sABI).

**Objective:**

This study aimed to estimate HAI incidence in different sABI rehabilitation settings and determine risk factors and HAI impact on neuromotor and cognitive recovery.

**Methods:**

We conducted a retrospective multicenter study in two semi-intensive units (SICUs), two high-specialty post-acute units (PAUs), and one long-term care (LTC) rehabilitation facility. Data extraction was performed by experienced clinicians, using a structured Excel file and they agreed upon criteria for case definitions of healthcare. The main outcome measures were the HAI and MDR HAI incidence and the LOS, the functional recovery was measured using the Level of Cognitive Functioning and Disability Rating Scale.

**Results:**

There were 134 sABI participants. The calculation of the probability level was adjusted for three pairwise comparisons among settings (0.05/3 = 0.017). The HAI and MDR HAI incidences were significantly higher in SICU (3.7 and 1.3 per 100 person-days) than in other settings (LTC: 1.9, *p* = 0.034 and 0.5, *p* = 0.026; PAU: 1.2, *p* < 0.001 and 0.3, *p* < 0.001). HAI and MDR HAI risk variables included older age, an increased number of devices, and carbapenemase-producing Enterobacteriaceae (CPE) colonization, while a high prealbumin plasma value seemed to have a protective effect.

**Conclusion:**

HAIs are related to longer LOS, and colonization is associated with poor prognosis and poor functional outcomes with reduced ability to achieve the cognitive capacity of self-care, employability, and independent living. The need to ensure the protection of non-colonized patients, especially those with severe disabilities on admission, is highlighted.

## Introduction

Healthcare-associated infections (HAIs) are a severe threat to patient safety in Europe. The European Center for Disease Prevention and Control (ECDC) surveys estimated an HAI prevalence of 6.5% in acute care hospitals and 3.9% in long-term care facilities (1). Patients hospitalized with a neurological diagnosis, especially those with severe acquired brain injury (sABI), are more likely to develop HAI (2) because of their immunodeficiency. It has only recently been realized that the impairment of the relationship between the central nervous system and the immune system caused by injury leads to secondary immunodeficiency (CNS injury-induced immunodepression, CIDS) and infection (3). In addition, medical devices, mental health problems, severe clinical conditions, and poor nutritional status (4) may contribute to further impairing their immune defenses.

Recent studies underscore that HAI and multidrug-resistant (MDR) HAIs affect rehabilitation outcomes and the hospital length of stay (LOS) for sABI patients (5). Research on HAIs and MDR HAIs, especially in rehabilitation and neurorehabilitation settings, is scant (6). The HAI prevalence among patients admitted to Rehabilitation Units in Europe has been reported to be approximately 8 to 15% (7). Respiratory tract infections, urinary tract infections (UTIs), ventilator-associated pneumonia, surgical site infections, surgical procedure-related infections, meningitis, and sepsis are common in intensive care units, especially if the length of stay exceeds 6 days (8). A recent study focused on MDR organism (MDRO) management in European rehabilitation facilities through a questionnaire. The prevalence is high, and the management of MDRO colonization is variable without any MDRO screening protocol, despite the increasing prevalence of CPE in healthcare facilities across Europe (9). An MDR pathogen organism is defined as non-susceptibility to at least one agent in three or more antimicrobial categories (10). The most common infections in this population are predominantly due to multiple pathogen germs and MDR germs, in particular: *Acinetobacter baumannii*, Enterobacteriaceae, *Pseudomonas aeruginosa*, and *Staphylococcus aureus* (11).

Data on the impact of carbapenemase-producing Enterobacteriaceae (CPE) colonization, which is the main risk factor for severe infection and high environmental spread (12), are also lacking.

The primary aim of the study was to estimate the HAI incidence and their etiology, distinguishing MDR and non-MDR infections, in sABI patients treated in inpatient rehabilitation settings such as semi-intensive care units (SICU), post-acute units (PAU), and long-term care (LTC) facilities. The secondary aims were to identify potential risk factors for HAI development and estimate the impact of HAIs on patients' recovery and LOS.

## Methods

### Study design and setting

This observational, retrospective multicenter study was conducted in four hospitals that comprise five different rehabilitation settings: two SICUs, two high-specialty PAUs, and one LTC facility. These wards are part of a clinical care pathway for sABI patients, encompassing the acute and rehabilitation phases up to discharge at home or to community facilities. All those settings are subjected to similar prevention and infection control measures according to the clinical care pathway. The two SICUs interact with the intensive care unit and neurosurgery to ensure a timely neurorehabilitation approach. They define the diagnosis, begin the rehabilitation process, and once patients are stabilized, they are directed to the next level of care. The two PAUs provide comprehensive care for sABI patients, offering separate units for disorders of consciousness with a long-term rehabilitation process. Based on the clinical stability and recovery of the patients, the trajectories of the rehabilitation path could differ. The possible trajectories were only SICU → PAU (*N* = 49), SICU → LTC (*N* = 2), SICU → PAU → LTC (*N* = 1), and PAU → LTC (*N* = 1). Unique trajectories were only SICU (*N* = 22), PAU (*N* = 39), and LTC (*N* = 20).

Patients not eligible for intensive rehabilitation treatment are directed to the LTC (13). All five wards use the same diagnostic and treatment approach with an individually customized rehabilitation plan that also involves the patients' families.

The study was approved by the local Ethics Committee (protocol No. 609-2019-OSS-AUSLIM) and did not receive funding. Informed consent was obtained when possible or waived in accordance with the General Authorization of the Privacy Guarantor No. 09/2016 on observational retrospective studies.

### Participants

Data on patients hospitalized in 2018, including patients admitted in 2017 and discharged by 31 December 2018, were extracted from medical records.

Data extraction was made by experienced clinicians, including some of the authors (GBC, AB, and EB), who entered the data extracted in a structured Excel file, using agreed criteria on case definitions of healthcare-associated infections based on ECDC Codebook (14) and are summarized in Supplementary Table S1.

During the study, some patients were admitted to more than one setting. In these cases, data were collected from both settings. Inclusion criteria were as follows: age ≥18 years, sABI of any etiological origin (traumatic, non-traumatic hemorrhagic, anoxic, infective, neoplastic, or toxic-metabolic), Glasgow Coma Scale ≤ 8 for at least 24 h, and impairments of physical, neurocognitive, and/or psychological function that involve a severe disability.

### Infection diagnosis

The number and etiology of infections occurring during hospitalization were collected. The diagnostic criteria of HAI were based on the ECDC Codebook (14) and are summarized in Supplementary Table S1. The most common infections are bloodstream infections (BSIs), UTIs, and pneumonia.

Other infections were considered if reported in medical records. They included *Clostridium difficile* infection, otitis, cellulitis, ventriculitis, phlebitis, epididymo-orchitis, skin and soft tissue infections, and intra-abdominal infections.

We omitted fewer common infections.

Infections were classified as non-MDR HAI or MDR HAI according to the organism found and based on the international expert proposal of Magiorako et al. (10) study.

### Outcome variables

The primary outcome was the incidence of HAIs and MDR HAIs. Secondary outcomes included LOS and functional recovery measured using the Disability Rating Scale (DRS) (15) and the Level of Cognitive Functioning Scale (LCF) (16), which are routinely administered in SICU and PAU settings. These functional assessment scales were not administered in the LTC setting.

LCF is a well-established tool to assess cognitive functioning in post-coma patients, validated in an Italian study (17). Patients are classified into eight levels, from 1 (non-responders) to 8 (purposeful-appropriate person); the higher the value, the better the cognitive function.

DRS is a 30-point scale measuring eight areas of functioning: eye-opening, verbalization, motor response, level of cognitive ability for daily activities of feeding, toileting, and grooming, overall level of dependence, and employability (18). Scores in each area (rated from 0 to 3 or 0 to 4 or 0 to 5) are summed to yield a total score between 0 and 30, with a higher score denoting lower functioning.

### Independent variables

The predictors of HAIs were classified into pre-admission characteristics, inpatient indicators, clinical parameters, and device-related data. Pre-admission variables were socio-demographic characteristics, history of previous diseases, type of injury, and any previous surgery. We added the condition of associated trauma for traumatic patients (Table 1) for those who suffered other traumatic lesions such as thoracic, skeletal, abdominal, or spinal cord. We also collected comorbidities, meaning specifically the presence of coexisting or additional pathologies with respect to brain damage with an infectious impact.

**Table 1:** Characteristics of the study sample at baseline and by rehabilitation setting.

**Variables**	**Study sample at admission *N =* 134**	**SICU *N =* 90**	**PAU *N =* 74**	**LTC *N =* 24**
Male, *n* (%)	89 (66.4)	60 (66.7)	52 (70.3)	13 (54.2)
Female, *n* (%)	45 (33.3)			
Age, mean ± SD	53.3 ± 18.2	51.1 ± 18.6	50.6 ± 16.1	69.8 ± 14.4
**Etiology**, ***n*** **(%)**				
Traumatic	54 (40.3)	39 (43.3)	33 (44.6)	4 (16.7)
Associated trauma^*^	21 (38.9)	33 (84.6)	27 (81.8)	1 (25.0)
Non-traumatic hemorragic	68 (50.7)	32 (35.6)	30 (40.5)	11 (45.8)
Other	12 (9.0)	19 (21.1)	11 (14.9)	9 (37.5)
Time since injury (days), median [IQR]	34.5 [18.7–79]	28 [15–45]	62.5 [35–94]	106 [28–148]
Comorbidity, *n* (%)	33 (24.6)	17 (18.9)	13 (17.6)	15 (62.5)
**Pre-admission surgery**, ***n*** **(%)**				
No	79 (59.0)	58 (64.4)	30 (40.5)	13 (54.2)
Neurosurgery	39 (29.1)	20 (22.2)	31 (41.9)	8 (33.3)
Other	16 (11.9)	12 (13.3)	13 (17.6)	3 (12.5)
LCF upon admission, median [IQR]	3 [2.25–5]	4 [3–5]	5 [3–6]	-
LCF at discharge, median [IQR]		5 [4–6]	6 [5–7]	-
DRS upon admission, mean ± SD	19 [16–23]	19 [15–22]	18 [14–20]	-
DRS at discharge, mean ± SD		16 [7–20]	11 [6–17]	-
Proteins (g/dL) upon admission	6.4 ± 1.4	6.5 ± 1.5	6.4 ± 0.8	6.2 ± 1.3
Albumin (g/L) upon admission	25.9 ± 11.9	31.1 ± 5.0	32.4 ± 5.7	28.3 ± 5.9
Prealbumin (mg/dL) upon admission	17.9 ± 6.8	17.9 ± 6.4	19.9 ± 6.3	17.2 ± 7.9
Transfers, *n* (%)		12 (13.3)	22 (29.7)	2 (8.3)
**Surgery during hospitalization**, ***n*** **(%)**				
No	113 (84.3)	83 (92.2)	54 (73.0)	22 (91.7)
Neurosurgery	11 (8.2)	2 (2.2)	16 (21.6)	0
Other	10 (7.5)	5 (5.6)	4 (5.4)	2 (8.3)
Ward shift caused by worsening, *n* (%)		6 (6.7)	8 (10.8)	0
**CPE colonization**, ***n*** **(%)**				
No	84 (62.7)	66 (73.3)	40 (54.0)	12 (50.0)
Upon admission	29 (21.6)	14 (15.6)	22 (29.7)	7 (29.2)
During hospitalization	21 (15.7)	10 (11.1)	12 (16.2)	5 (20.8)
Complications, *n* (%)	48 (35.8)	10 (11.1)	46 (62.2)	23 (95.8)
Length of stay (days), median [IQR]		20.5 [13–42]	103 [55–203]	47.5 [9–169]
**Medical devices**				
Indwelling catheter, *n* (%) days, median [IQ range]		78 (86.7) 21.5 [10–55]	44 (59.5) 55 [19–101]	24 (100) 43.5 [9–156]
Central venous catheter, *n* (%) days, median [IQ range]		40 (44.4) 14 [7–33]	18 (24.3) 24.5 [6–53]	3 (12.5) 45 [8–164]
Tracheostomy tube, *n* (%) days, median [IQ range]		65 (72.2) 32 [17–70]	46 (62.2) 99 [30–200]	13 (54.2) 42 [10–148]
PEG or PEJ, *n* (%) days, median [IQ range]		29 (32.2) 44 [17–76]	32 (43.2) 225 [110–285]	13 (54.2) 98 [42–173]

^*^Among patients with traumatic etiology; continuous data are reported as mean ± SD or median and [interquartile range, IQR]; categorical variables are reported as count (n) and column percentages (%). SICU, semi-intensive care unit; PAU, post-acute unit; LTC, long-term care facility; LCF, levels of cognitive functioning; DRS, disability rating scale; CPE, carbapenemase-producing Enterobacteriaceae; PEG, percutaneous endoscopic gastrostomy.

Inpatient indicators included the time between injury and admission, any transfers to other departments, any surgery during hospitalization, and any ward shifts caused by clinical worsening.

Clinical parameters were CPE colonization on admission or during rehabilitation and nutritional parameters (protein, albumin, and prealbumin assay) on admission. Moreover, clinical conditions with a potential negative impact on the functional outcome not directly related to HAIs (cardiovascular complications, osteoarticular problems, wound onset, and dystonia) were collected.

Devices included an indwelling urinary catheter, a central venous catheter, a tracheotomy tube, and a percutaneous endoscopic gastrostomy. The number of medical devices used for each patient was collected.

### Statistical methods

Continuous variables were summarized using mean and standard deviation (±SD) when normally distributed, and median and interquartile range [IQR] otherwise; categorical variables were summarized using frequencies.

The incidence of HAIs and MDR HAIs per 100 person-days was calculated using the length of hospital stay as the exposure time. HAI incidence was compared between the three settings. In case of a significant difference, the rehabilitation setting was included as an adjustment variable in the subsequent analyses.

A negative binomial regression analysis was used to identify the risk factors associated with HAI and MDR HAI incidence. Significant variables were included in a multivariable model. The results were reported as an incidence rate ratio (IRR) and 95% confidence interval (95% CI). All the significance levels reported refer to comparisons of regression coefficients in the Poisson and negative binomial models. When more than two groups were compared, a Bonferroni correction to the probability level was applied. The adjustment for the rehabilitation setting is obtained by including the rehabilitation setting in the model as two dummy variables, one for PAU and one for LTC, and using SICU as the reference category.

In a secondary analysis, including data from SICU and PAU settings only, patients were classified into three mutually exclusive groups: no infection, at least one HAI (non-MDR), and at least one MDR HAI.

These groups were compared on LOS and the rehabilitation outcomes (DRS and LCF scores at discharge), using negative binomial regression and linear regression, respectively. The results on LOS were reported as predicted LOS (in weeks) with 95%CI. In linear regression analysis, a model adjusted for rehabilitation setting and functional score on admission was initially estimated, then a multivariable model adjusted for other factors was obtained. Regression coefficients were reported with 95%CI.

The backward stepwise variable selection procedure was applied to obtain parsimonious multivariable models (p for removal=0.05). Robust standard errors were estimated using a clustered sandwich estimator to take into account repeated measurements (i.e., admissions in different settings) on the same individual. Bonferroni's correction was applied for multiple comparisons. The Bonferroni correction consists of adopting a significance level adjusted for the number of comparisons when the groups compared are more than 2.

In this case, the probability level was adjusted for three pairwise comparisons among settings (0.05/3 = 0.017). The statistical software (Stata), when the Bonferroni correction is requested, provides already adjusted *p*-values. For instance, if the *p*-value of a test is 0.002, it is provided in the output as 0.002^*^3 = 0.006. All the *p*-values in our results are adjusted *p*-values when they refer to comparisons of the three settings.

Statistical analyses were carried out using Stata version 15 (StataCorp, College Station, TX). The significance level was set to a *p*-value of < 0.05.

## Results

### Patients' characteristics

The analysis included 188 records of 134 patients admitted to at least one of the four hospitals. During the study period, 49 patients were transferred from SICU to PAU, 2 patients from SICU to LTC, 1 patient from PAU to LTC, and 1 patient crossed all three settings (Supplementary Figure S1). Overall, the analysis included the data of 90 patients hospitalized in SICU, 74 patients hospitalized in PAU, and 24 patients hospitalized in LTC. Patients' characteristics by setting are reported in Table 1. The mean age was 53.3 ±18.2 years, and 66.5% of patients were male. Traumatic etiology accounted for 40.4% of cases, and 23.9% of patients had comorbid conditions. In LTC, the sample was older on average, had more comorbidities, and had a lower frequency of traumatic etiology. The median LOS was 21 days [13–42] in SICU, 103 days [55–203] in PAU, and 48 days [9–169] in LTC.

### HAI incidence

The HAI incidence ranged from 1.2 per 100 person-days in PAU to 1.9 in LTC and up to 3.7 in SICU (Table 2). The incidence in SICU was significantly higher than in the other settings (*p* < 0.001 SICU vs. PAU; *p* = 0.034 SICU vs. LTC). MDR HAI incidence was 0.3 per 100 person-days in PAU, 0.5 in LTC, and 1.3 in SICU. The incidence in SICU was significantly higher compared to the other two settings (*p* < 0.001 SICU vs. PAU; *p* = 0.026 SICU vs. LTC).

**Table 2:** HAI and MDR HAI incidence in each rehabilitation setting.

	**Num. HAI**	**HAI incidence per 100 person-days**	**95%CI**		**Num. MDR HAI**	**MDR HAI incidence per 100 person-days**	**95%CI**
SICU	115	3.74	2.50–4.99		41	1.33	0.82–1.85
PAU	122	1.23	0.92–1.55		32	0.32	0.17–0.48
LTC	43	1.93	1.03–2.83		12	0.54	0.20–0.88

SICU, semi-intensive care unit; PAU, post-acute unit; LTC, long-term care facility; HAI, healthcare-associated infections; MDR, multidrug-resistant.

Supplementary Figure S2 reports the HAI and MDR HAI incidence in each rehabilitation setting, according to the infection etiology. Overall and MDR bloodstream infections (BSIs) had a significantly higher incidence in the SICU as compared to the other settings. The overall UTI incidence was higher in SICU (0.68, 95%CI: 0.33–1.03) than in LTC (0.45, 95%CI: 0.11–0.78) and PAU (0.20, 95%CI: 0.10–0.31), but only the difference between SICU and PAU was significant (*p* = 0.017). On the other hand, the overall incidence of pneumonia was similar in SICU (1.01, 95%CI: 0.46–1.55) and LTC (0.94, 95%CI: 0.37–1.52), while it was significantly lower in PAU (0.36, 95%CI: 0.21–0.52). MDR pneumonia incidence had a similar trend. A low incidence of MDR UTIs, skin and soft tissue infections, and intra-abdominal infections was observed in all three settings. The incidence of other infections was higher in SICU as compared to the other two settings. Overall, 280 infections occurred; the most frequent pathogens involved were *P. aeruginosa* (15.7%), *K. pneumoniae* (14.3%), *E. coli* (10%), *P. mirabilis* (7.4%), *Candida* spp. (4.8%), *E. faecalis* (4.3%), and *A. baumannii* (3.9%). In 26.1% of cases, the pathogen was not identified.

Among the 280 HAIs, 85 were identified as MDR (30.3%). The MDR frequency on certain isolates was higher than 50%, specifically: *K. pneumoniae* (55%, *N* = 33), *A. baumannii* (89%, *N* = 9), methicillin-resistant *S. epidermidis*, MRSE (*N* = 8), methicillin-resistant *S. aureus*, MRSA (*N* = 6), and *E. cloacae* (100%, *N* = 4).

### Factors associated with HAI incidence

Table 3 shows the factors associated with HAI and MDR HAI incidence. In the regression analysis adjusting for rehabilitation setting only, older age, any comorbidities, lower LCF and higher DRS scores upon admission, higher number of devices, and CPE colonization were all significantly associated with HAI development during inpatient rehabilitation. Conversely, a high prealbumin plasma value on admission was associated with a decreased likelihood of developing HAI. In the multiple regression analysis, older age, an increased number of devices, and CPE colonization remained significant. Older age and CPE colonization were also significant risk factors for MDR HAI incidence.

**Table 3:** Factors associated with HAI and MDR HAI incidence: results of bivariate and multiple negative binomial regression models, adjusted for rehabilitation setting.

	**Overall HAI**				**MDR HAI**			
	**Bivariate model**		**Multiple model**		**Bivariate model**		**Multiple model**	
	**IRR (95%CI)**	**p-value**	**IRR (95%CI)**	**p-value**	**IRR (95%CI)**	**p-value**	**IRR (95%CI)**	**p-value**
Male	0.91 (0.60–1.38)	0.653			1.27 (0.63–2.55)	0.508		
Age	1.02 (1.01–1.03)	**< 0.001**	1.02 (1.01–1.03)	**0.006**	1.03 (1.01–1.05)	**0.010**	1.03 (1.00–1.05)	**0.042**
**Etiology** Traumatic (ref.) Vascular Other	1.00 1.40 (0.91–2.16) 1.44 (0.95–2.20)	0.208			1.00 1.22 (0.56–2.65) 2.03 (0.91–4.52)	0.263		
**Pre-admission surgery** No (ref.) Neurosurgery Other	1.00 0.85 (0.58–1.26) 0.91 (0.48–1.74)	0.753			1.00 0.79 (0.41–1.52) 1.00 (0.32–3.09)	0.805		
Comorbidity	1.69 (1.16–2.47)	**0.007**			1.48 (0.76–2.88)	0.249		
LCF upon admission	0.85 (0.75–0.96)	**0.010**			0.79 (0.65–0.96)	**0.016**		
DRS upon admission	1.05 (1.02–1.09)	**0.002**			1.09 (1.02–1.16)	**0.008**		
Proteins upon admission	0.94 (0.78–1.13)	0.511			0.85 (0.63–1.15)	0.303		
Albumin upon admission	0.98 (0.94–1.03)	0.381			0.95 (0.88–1.03)	0.199		
Prealbumin upon admission	0.96 (0.93–0.99)	**0.011**			0.93 (0.86–0.99)	**0.029**		
Number of medical devices	1.51 (1.28–1.79)	**< 0.001**	1.34 (1.15–1.55)	**< 0.001**	1.33 (0.98–1.81)	0.065		
CPE colonization	2.14 (1.45–3.15)	**< 0.001**	1.65 (1.09–2.48)	**0.017**	4.20 (1.89–9.33)	**< 0.001**	3.78 (1.67–8.58)	**0.001**
Transfers	0.81 (0.55–1.19)	0.286			0.86 (0.44–1.67)	0.655		
**Surgery during hospitalization**	0.72 (0.47–1.09)	0.121			0.61 (0.26–1.41)	0.247		
Ward shift caused by worsening	1.23 (0.77–1.96)	0.385			2.10 (1.08–4.09)	**0.029**		
Complications	1.29 (0.83–1.99)	0.254			1.04 (0.50–2.18)	0.906		

Nutritional parameters, baseline LCF, and baseline DRS scores were excluded from multiple model estimation because of missing data; significant factors are given in bold. HAI, healthcare-associated infections; MDR, multidrug-resistant; LCF, levels of cognitive functioning; DRS, disability rating scale; CPE, carbapenemase-producing Enterobacteriaceae.

### Association between HAI and rehabilitation outcomes

**Figure 1:**
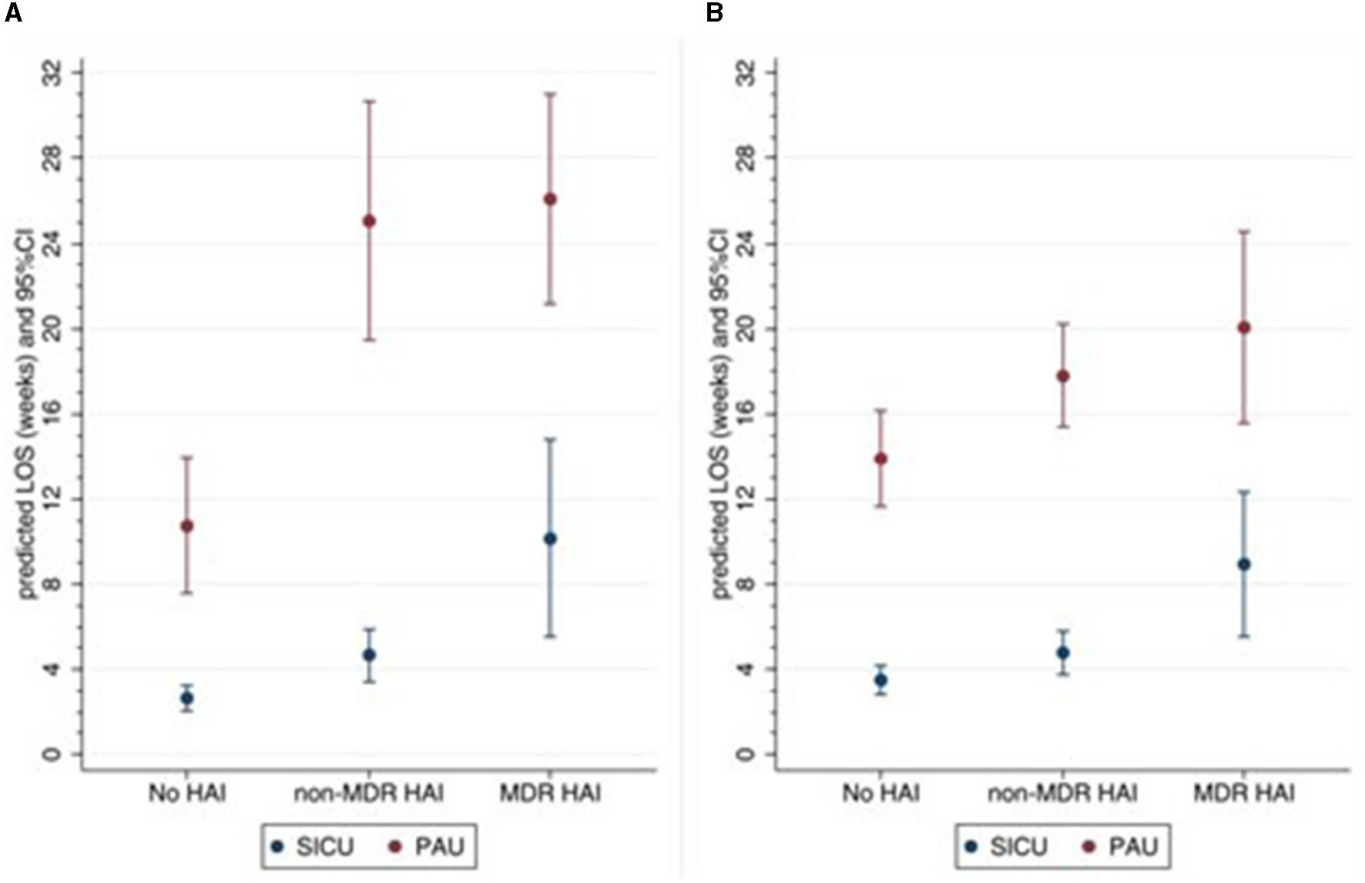
Predicted LOS according to setting and HAI groups. **(A)** Unadjusted, **(B)** adjusted for number of medical devices, surgery during rehabilitation, any complications. HAI, healthcare-associated infections; MDR, multidrug-resistant; SICU, semi-intensive care unit; PAU, post-acute unit; LOS length of stay.

In the SICU setting, LOS was significantly higher in the HAI group compared with the no-infection group (4.7 vs. 2.6 weeks, *p* = 0.008) and even higher in the MDR HAI group (10.2 weeks, *p* = 0.003) (Figure 1A). In the PAU setting, LOS was on average 11 weeks in the no-infection group and approximately 25–26 weeks in the HAI and MDR HAI groups. After adjusting for the number of devices, surgery during rehabilitation, and complications, the differences between the three groups remained significant (Figure 1B).

In a regression model adjusted for admission score and rehabilitation setting, being in the MDR HAIs group was significantly associated with higher DRS and lower LCF scores at discharge (Table 4). However, these associations were no longer significant after adjusting for the number of devices, surgery during rehabilitation, and complications. Notably, in the presence of CPE colonization and complications, disability at discharge increased as the time from injury to hospitalization increased. Similarly, the LCF score at discharge was lower for patients with an increased time from injury to hospitalization, patients who underwent neurosurgery before rehabilitation, and those with an etiology other than traumatic or vascular.

**Table 4:** Association between HAI groups and DRS and LCF scores at discharge: results from multiple linear regressions.

	**DRS**				**LCF**			
	**Model 1**		**Model 2**		**Model 1**		**Model 2**	
	**b (95%CI)**	**p-value**	**b (95%CI)**	**p-value**	**b (95%CI)**	**p-value**	**b (95%CI)**	**p-value**
**HAI groups** No HAI (ref.) HAI non–MDR HAI MDR	– 0.30 (−1.25–1.84) 2.35 (0.61–4.09)	0.703 0.009	– −0.59 (−2.06–0.89) 0.28 (−1.91–2.47)	0.432 0.802	– −0.14 (−0.50–0.22) −0.58 (−1.08 to −0.09)	0.430 0.022	– 0.04 (−0.32–0.40) −0.20 (−0.75–0.35)	0.825 0.478
Score upon admission	0.93 (0.83–1.02)	< 0.001	0.90 (0.80–1.00)	< 0.001	0.82 (0.72–0.93)	< 0.001	0.75 (0.65–0.86)	< 0.001
SICU (ref.) PAU	– −2.22 (−3.58 to −0.87)	0.001	– −3.66 (−5.05 to −2.28)	< 0.001	0.49 (0.16–0.82)	0.004	0.65 (0.34–0.97)	< 0.001
Time between injury and rehabilitation (weeks)			0.08 (0.03–0.12)	0.001			−0.02 (−0.04 to −0.01)	0.002
CPE colonization			1.88 (0.33–3.45)	0.018				
Any complication			1.54 (0.17–2.92)	0.028				
**Pre–admission surgery** No (ref.) Neurosurgery Other							– −0.50 (−0.88 to −0.13) 0.06 (−0.32–0.45)	0.008 0.741
**Etiology** Traumatic (ref.) Vascular Other							– 0.06 (−0.31–0.42) −0.74 (−1.24 to −0.24)	0.757 0.004

Model 1 includes HAI groups, the score at admission, and the rehabilitation setting; Model 2 includes, in addition, other factors associated with the score at discharge. HAI, healthcare-associated infections; MDR, multidrug-resistant; LCF, levels of cognitive functioning; DRS, disability rating scale; SICU, semi-intensive care unit; PAU, post-acute unit; CPE, carbapenemase-producing Enterobacteriaceae.

## Discussion

In this study, we investigated and compared HAI incidences among sABI patients in different settings. We found that in SICU HAIs and MDR HAIs, the incidence was higher than in other settings, especially for BSIs. Pneumonia and UTIs were also more frequent in SICU and LTC settings than in PAU.

According to the ECDC and a previous study (8), intensive care units are the hospital wards with the highest prevalence of HAIs associated with the use of invasive devices and prolonged hospitalization. Moreover, in the intensive care unit setting, the burden of antimicrobial resistance is high due to the severity of the clinical condition of the patients, the frequent use of antibiotics, and varying infection prevention and control practices (19). In addition, it is established that central nervous system injury is an independent risk factor for increased susceptibility to infections (20). In fact, injury leads to secondary immunodeficiency, causing a disturbance of the interplay between the immune system and the central nervous system (3). Dziedzic et al. (20) investigated the clinical significance of the immune status in the development of nosocomial infections in brain-injured patients, where the critical abnormalities include an overall reduction in helper and regulatory T-cell frequencies, reduced proliferation of cytotoxic T cells, reductions in NK and B cell numbers, and increased production of IL-6 and IL-10 from monocytes.

Interestingly, as underscored by Meisel et al. (3), the predominantly proinflammatory response in the CNS is in contrast to the well-organized anti-inflammatory response by the peripheral system. Injury-induced compensatory anti-inflammatory response system may prove to be beneficial as it could control excessive systemic inflammation; however, this response may also be triggered in the absence of a systemic stimulus, i.e., in the case of a TBI, resulting in a detrimental anti-inflammatory dominant response that causes systemic immune system shutdown. This involves the release of immunomodulators from the injured brain into the circulation, which in turn instigates a state of imbalance between pro- and anti-inflammatory mediator cascades, which further weakens the systemic immune defense system. For these reasons, it would be appropriate to increase the attention and control level during the earliest stages of rehabilitation when the patient is more fragile and subjected to invasive devices, as we found in the SICU setting. Close attention is required to adhere to hand hygiene and contact precautions.

The secondary goals of the study included the identification of potential risk factors for HAI development. We found that higher age, CPE colonization, and a higher number of devices are significant risk factors for HAI, with the first two also being significant factors for MDR HAI. Conversely, a high prealbumin plasma value on admission was associated with a lower risk of HAI and MDR HAI occurrence.

Elderly patients are at high risk of HAIs due to the age-related decline of the immune system, known as immunosenescence. Comorbid conditions can often complicate infections, diminishing the ability to treat them effectively (21). We included all the comorbidities with an infectious impact, and we observed that cardiologic and endocrine-metabolic were the most frequent, according to Bellaviti et al. (5) study.

Devices predispose to infection by damaging or invading epithelial and mucosal barriers and by supporting the growth of biofilms implicated in the development of medical device-related infections (22). sABI patients frequently require several intensive therapy measures that involve devices, such as tracheotomy with assisted breathing, bladder catheterization, and parenteral nutrition.

As to CPE colonization, previous studies found a peculiar CPE epidemiology in long-term acute rehabilitation facilities with high rates of cross-transmission in sABI patients (23). CPE is a dangerous MDRO because most of the carbapenemase-encoding genes are located on transferable genetic elements associated with other antibiotic resistance genes, leading to their rapid transfer and the spread of uncontrollable superbugs (24). Indeed, CPE is among the major causative agents of nosocomial infections (25). In Europe, *K. pneumoniae* is a common cause of BSI, UTIs, and respiratory tract infections, and it is easily transmitted between patients, resulting in nosocomial outbreaks and high rates of morbidity and mortality, reaching 70% in some countries, with attributable mortality for BSI of 50% (26).

The protective effect of a high prealbumin value confirms the importance of nutritional status in contrast to MRDOs. Unfortunately, sABI patients can develop potential severe complications with high protein expenditures, such as pressure sores and muscular weakness, which significantly increase the risk of infection. Boselli et al. (4) reported that the supplementation of essential amino acids may reduce the occurrence of HAI in sABI patients and that low levels of prealbumin and high levels of c-reactive protein are predictors of infections (4). Another study identified serum albumin and prealbumin as predictors for unfavorable outcomes in traumatic brain injury, but in the subgroup of sABI patients, just serum albumin remained significant (27). We can assume, hence, that the prealbumin level can be used as a marker of frailty and that one goal of SICU and rehabilitation settings is to reach or maintain a good nutritional status in sABI patients.

Regarding the impact of HAIs on patient recovery, MDR HAIs seemed associated with a worse outcome (higher DRS and lower LCF), but when other factors (e.g., time to rehabilitation, CPE colonization, and complications) were taken into account, the association was no longer significant.

However, the study by Rollnik showed that in patients in early neurological rehabilitation, the improvement achieved was comparable between patients with and without MDROs (28). On the contrary, in a recent Italian study (29) on sABI patients admitted to three neurorehabilitation centers, those with infections showed a significantly lower improvement in physical function, a higher LOS, and a higher rate of mortality than subjects without infection or colonization.

In line with Bartolo et al. (6), our results indicate that CPE colonization was a significant risk factor for higher disability (higher DRS score) at discharge. This result highlights the importance of minimizing the risk of CPE colonization during inpatient rehabilitation. This can be done by means of appropriate infection prevention practices, such as hand hygiene, environmental cleaning, favoring single-room accommodations, the use of surveillance cultures to identify unrecognized carriers, contact precautions, and isolation, as well as enhanced antimicrobial stewardship to prevent the emergence of resistance. However, the implementation of infection prevention practices is particularly difficult in rehabilitation facilities because of the nature of inpatient care, which by definition is of a longer duration than that in acute care hospitals and in many cases may involve the facility becoming the patient's home environment (26). The availability of appropriate single rooms and MDRO screening appear to be a major infrastructural deficit (30) and the isolation of patients with MDROs presents a serious disadvantage for the rehabilitation outcome (31). The common prevention and infection control practices in our clinical care pathway might improve the management and safety of patients.

We found no significant difference between traumatic, vascular, or other etiologies with respect to the incidence of HAIs (Table 3). Therefore, we found a lower frequency of traumatic etiology in LTC, and the LCF score at discharge was lower for those who had an etiology other than traumatic or vascular.

Finally, we investigated the hospital LOS and found that patients with HAI had a longer LOS; moreover, in SICU, MDR HAI was associated with a higher LOS as compared to HAI. The significantly higher LOS for infected patients was also reported in other studies (5, 6).

Taken together, the results on rehabilitation outcome and LOS indicate that in our clinical pathway, HAI impact on rehabilitation determines a prolonged LOS rather than a worse outcome at discharge. The absence of association between infections and outcomes can be explained by considering that the clinical pathway aims at discharging patients once their best possible outcome is reached, based on personalized goals.

Furthermore, the relationship between infections and LOS can also be interpreted in the opposite direction [34]: As the length of hospitalization increases, the risk of infection increases. Thus, clinicians need to direct their efforts to minimize hospital LOS, taking all the possible preventive measures to reduce the risk and rate of infection on the one hand and improving the efficiency of the rehabilitation process to reduce the risk of complications on the other. This multifactorial approach can improve the health of patients and the costs of the healthcare system.

## Limitations

Due to the retrospective study design, information on nutritional parameters and LCF and DRS scales was missing or not available in the LTC setting, and the identification of infectious events was sometimes difficult. Indeed, data were retrieved from clinical records, and some information on infections was inferred from antibiotic therapies, requests for consultation with an infectious disease specialist, and any other report related to infection. As a result, the infection rate could have been underestimated.

## Conclusion

Our study suggests that the management of HAIs and antimicrobial resistance risk is crucial to exploiting the potential for recovery of sABI across all stages of the rehabilitation pathway. Neglecting or underestimating this problem may delay or prolong the rehabilitation process and frustrate patients, caregivers, and healthcare professionals' efforts. The key finding is that HAIs are related to longer LOS, and colonization is associated with poor prognosis and outcomes. Hence, we underscore the need to ensure the protection of non-colonized patients, especially those with severe disabilities on admission.

## Data availability statement

The original contributions presented in the study are included in the article/Supplementary material, further inquiries can be directed to the corresponding author.

## Author contributions

GC, EB, and AB contributed to conception and design of the study. EM and GL organized the database. EM performed the statistical analysis. GC, EM, and GL wrote the first draft of the manuscript. EB, AB, ST, and FT wrote sections of the manuscript. RP and PV reviewed the final version. All authors contributed to manuscript revision, read and approved the submitted version.

## In memoriam

During the preparation of this paper Dr. Elisa Maietti prematurely died. She was a brilliant statistician and an unforgettable joyful, radiant, generous and supportive colleague. This paper is dedicated to her memory.
