# Supplementary material for: Healthcare-associated infections and antimicrobial resistance in severe acquired brain injury: a retrospective multicenter study
Source: Front Neurol. 2023 Aug 16;14:1219862. doi: 10.3389/fneur.2023.1219862 (PMC10469002; doi:10.3389/fneur.2023.1219862)
Supplement: Supplementary file 1 [file Table_1.pdf]

Table S1. Case definitions of healthcare associated infections (PPS of HAIs and antimicrobial use in European acute care hospitals – protocol version 5.3. Stockholm: ECDC; 2016).

HAI	DIAGNOSTIC CRITERIA		
Pneumonia	chest X-rays or CT-scans images suggestive of pneumonia	and fever > 38 °C with no other cause<br>or<br>leukopenia or leukocytosis<br>plus<br>at least one of the following:<br>• new onset of purulent sputum or tracheal secretions;<br>• cough or dyspnea or tachypnea;<br>• suggestive auscultation, rhonchi, wheezing;<br>• worsening gas exchange	and according to positive quantitative culture from minimally or possibly contaminated Lower Respiratory Tract specimen.
Urinary Tract Infection		at least one of the following signs or symptoms with no other recognized cause:<br>• fever (> 38°C),<br>• urgency,<br>• frequency,<br>• dysuria,<br>• suprapubic tenderness	and a positive urine culture, that is, ≥ 10^5 microorganisms per ml of urine with no more than two species of microorganisms.
Bloodstream Infection			one positive blood culture for a recognized pathogen
		or at least one of the following signs or symptoms:<br>• fever (> 38°C),<br>• chills,<br>• hypotension	and two positive blood cultures for a common skin contaminant (from two separate blood samples, usually within 48 hours).
Skin and Soft Tissue Infection		at least one of the following criteria:<br>purulent drainage,<br>pustules,<br>vesicles,<br>boils	
		or, at least, two of the following signs or symptoms with no other recognized cause:<br>• pain or tenderness,<br>• localized swelling,<br>• redness,<br>• heat	and at least one of the following: organisms cultured from aspirate drainage from affected site<br>or organisms cultured from blood
Intra-abdominal Infections			organisms cultured from purulent material from intra-abdominal space obtained during a surgical operation or needle aspiration; or abscess or other evidence of intra-abdominal infection seen during a surgical operation or histopathologic examination
		or at least two of the following signs or symptoms with no other recognized causes:<br>• fever (> 38 °C),<br>• nausea,<br>• vomiting,<br>• abdominal pain,<br>• jaundice	and at least one of the following:<br>• organisms cultured from drainage from surgically placed drain;<br>• organisms seen on Gram stain of drainage or tissue obtained during surgical operation or needle aspiration;<br>• organisms cultured from blood and radiographic evidence of infection, e.g. abnormal findings on ultrasound, CT scan, MRI, or radiolabel scans or on abdominal X-ray
