# Supplementary figures and images for: Healthcare-associated infections and antimicrobial resistance in severe acquired brain injury: a retrospective multicenter study
Source: Front Neurol. 2023 Aug 16;14:1219862. doi: 10.3389/fneur.2023.1219862 (PMC10469002; doi:10.3389/fneur.2023.1219862)

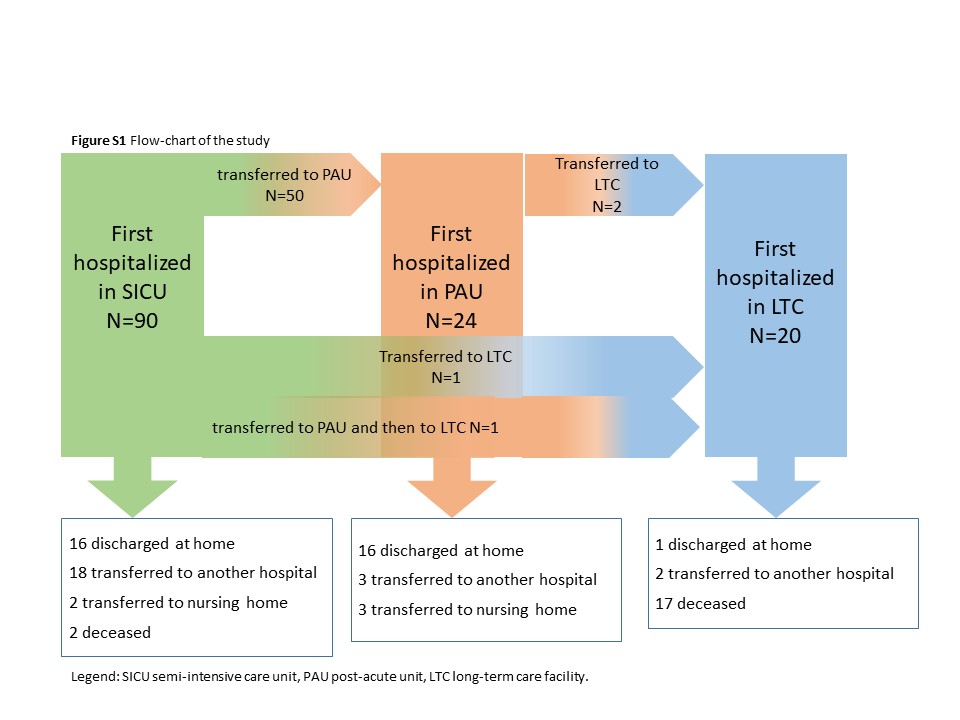

Supplement: Supplementary file 2 [file Image_1.jpg]

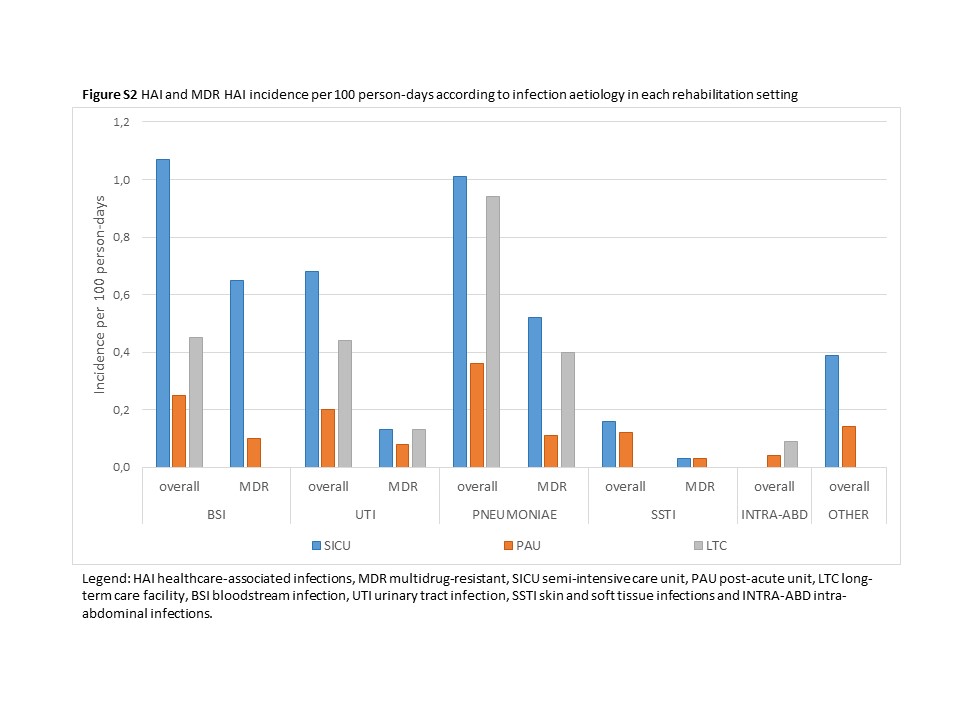

Supplement: Supplementary file 3 [file Image_2.jpg]
